# Scientific collaboration: a social network analysis based on literature of animal-derived regenerative implantable medical devices

**DOI:** 10.1093/rb/rbw019

**Published:** 2016-05-18

**Authors:** Shu-Yang Yu, Hong-Man Wang

**Affiliations:** Section of Medical Sociology and Medical Anthropology, Institute for Medical Humanities, Peking University Health Science Center, Beijing 100191, China

**Keywords:** scientific collaboration, tissue engineering, social network analysis, medical devices, ADRIMD

## Abstract

The collaboration network of English publications on animal-derived regenerative implantable medical devices based on tissue engineering technology and its evolving processes and current states were mapped in this paper. A total of 10 159 English papers published before 1 January 2015 were obtained in eight databases. Social network analysis was conducted on these papers by utilizing UCINET software and Statistical Analysis Software for Informatics researched and developed by Peking University. The collaboration network has evolved from scattered formation to single-core dominated, and then to a core-edge one; collaboration has become more frequent and wider; network density and centrality have decreased; USA, UK and China are the top three countries with Wake Forest University, Harvard University and Tufts University being the top three contributing institutions cooperated mostly during the period between 2010 and 2014; plenty of edge institutes exist. In conclusion, more collaboration among different institutions and countries is needed; Edge institutions and developing countries should expand their scope of collaboration.

## Introduction

The term tissue engineering was first coined in 1988 at a meeting of the US National Science Foundation. It refers to ‘an interdisciplinary field that combines knowledge and technology of cells, biomaterials as well as suitable biochemical factors to fabricate artificial organs, tissues or to regenerate damaged sites’ [1]. Tissue engineering is a promising new field of medical technologies, and it has been studied and applied to various organs [2]. With further development, it might alleviate suffering caused by tissue or organ damages and lead to longer and healthier lives [3].

In recent years, scientists around the world have conducted extensive research in the field of tissue engineering and this had led to notable discoveries and achievements on seed cells, scaffold materials and so on. Tissue engineering is an interdisciplinary science involving biology, medicine, materials science, engineering science, computer science as well as other relevant scientific fields. With the development of science and technology, collaboration between different departments becomes an important factor to increase research outputs. It provides not just a method of increasing knowledge and obtaining professional achievements, but it is also an effective way of acquiring scientific resources and establishing communication network for scientific elites [4].

New ways of measuring research are being proposed [5]. A relatively novel method for quantifying research output is social network analysis (SNA) [6]. SNA is concerning the relationships between social behaviors [7–9]. It is a method for mapping and measuring the relationships between papers, journals, researchers and institutions. There were many researchers conducted collaboration research by SNA. Morel *et al*. [10] used SNA to assess national and international collaborations of Germany-based researchers and research institutions working on five neglected tropical diseases. Long *et al*. [11] reported on a social network survey of the translational research network and focused on the structure of the collaborative arrangements among members. Okamoto [12] conducted a SNA of the centers for population health and health disparities. You *et al*. [13] focused on Chinese oncology drug research communities in co-publication networks at the institutional level and used SNA to define an institutions network and to identify a community network which was characterized by thematic content. Petrescu-Prahova *et al*. [14] examined the structure of mentorship and collaboration relationships among members of the healthy aging research network using SNA. Using data from the top 10 nursing journals in China from 2003 to 2013, Hou *et al*. [15] constructed a nursing scientific coauthor ship network using SNA. Uddin *et al*. [16] develop a research framework to explore health care coordination and collaboration by SNA. Wu and Duan [17] measured the activities of scientific collaboration in psychiatry research at the level of authors, institutions and countries by SNA. SNA which represents the connections between individuals can be valuable analytic tools [18]. 

Taking ADRIMD (animal-derived regenerative implantable medical devices) based on tissue engineering technology as an example; this paper analysed relevant publications in English to describe the collaboration status and evolution in this area by using SNA.

## Data and methods

### Data

The search strategy for identifying articles entailed 11 expressions: (biomaterial* AND regenerat*), (tissue engineer* AND regenerat*), (composite* AND regenerat*), (small intestinal submucosa AND regenerat*), (xenograft AND regenerat*), (xeno-implant* AND regenerat*), (heterogeneous graft* AND regenerat*), (acellular matrix AND regenerat*), (decellular matrix AND regenerat*), (acellular scaffold AND regenerat*) and (decellular scaffold AND regenerat*). Titles and abstracts of eight databases (PubMed, ScienceDirect, Web of Science, EBSCO, SpringerLink, Engineering Index, BIOSIS Preview and ProQuest Dissertations and Theses) were searched.

Since the search was carried out on 30 August 2015, the annual data for year 2015 are incomplete. We therefore selected all the results dated before 1 January 2015. There were a total of 16 352 records obtained. We excluded news, editorials, interviews, letters, books, non-English papers and others that we deemed irrelevant. This left us with 10 159 records. Irrelevant literature refers to the articles with literature focuses mainly on non-medical field (e.g. environmental science, materials for industry use), or concerns mainly based on synthetic scaffolds, plant-derived scaffolds autologous implants or allografts.

### Methods

SNA was used to analyse the collaboration in the field of ADRIMD. First, the institution information of authors was standardized, which meant the same institution coded with a uniform name, assigned to by authors. Then, SASI 1.0 with its copyright belongs to the Peking University (China), was used to calculate co-occurrence matrix, augmented matrix, net density and so on. The collaboration net was then visualized by UCINET 6.

Of note: SASI 1.0 was approved its copyright in 2015. Principle investigators are Hong-Man Wang and Shu-Yang Yu.

## Results

### Collaboration in general

**Figure 1:**
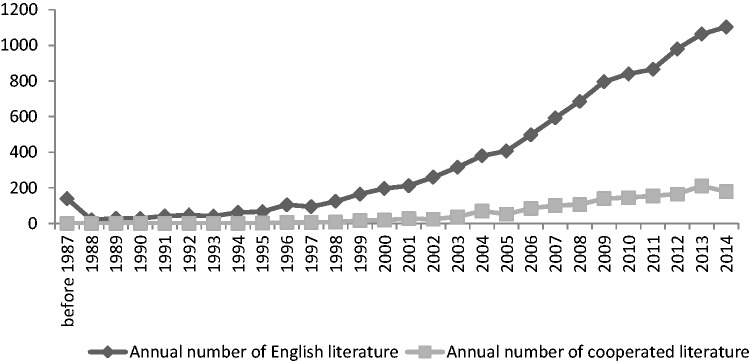
Annual number of English literature

There were a total of 10 159 publications in English by authors from 2104 institutions. The number of publications increased year by year (as shown in fig. 1). The literature finished collaboratively by multiple institutions has been of an increasing trend. But it is obvious that most of the literature was finished by single institution.

Top 10 institutions having the most number of cooperated papers are shown in Table 1. Most of them are universities. As for top 10 countries, most of them are developed countries (see Table 2).

**Table 1:** Top 10 institutions with cooperated papers

Institution	Number of cooperated paper
Harvard University (USA)	40
National University of Singapore (Singapore)	32
University of California (USA)	32
University of Pittsburgh (USA)	26
Shanghai Jiao Tong University (China)	26
Japan National Institute for Materials Science (Japan)	24
MIT (USA)	24
Tufts University (USA)	23
Wake Forest University (USA)	20
Tokyo Medical and Dental University (Japan)	20

**Table 2:** Top 10 countries with cooperated papers

Countries	Number of cooperated paper
USA	197
China	97
UK	79
Germany	73
Italy	49
Switzerland	42
Japan	39
Korea	32
Singapore	32
Australia	30

### Evolution of social collaboration network

**Figure 2:**
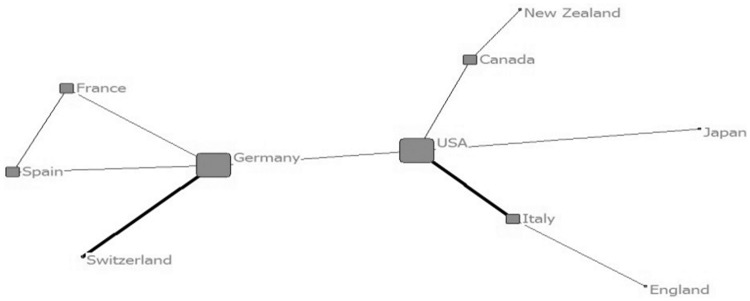
Collaboration among countries (before 1999)

**Figure 3:**
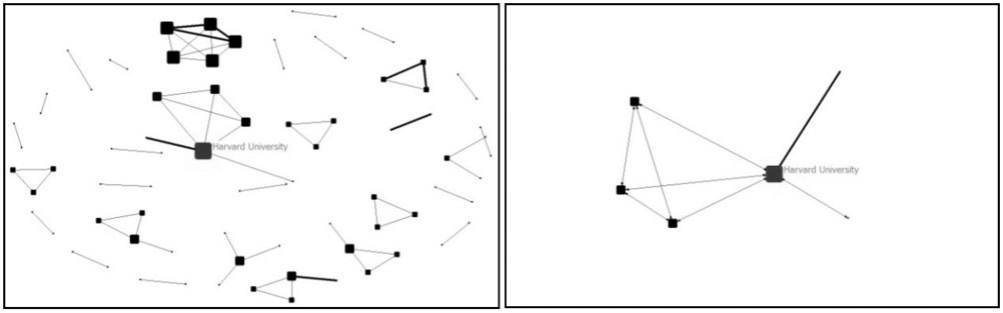
All institutions collaboration network (left) and the biggest collaboration team (right) (before 1999)

**Figure 4:**
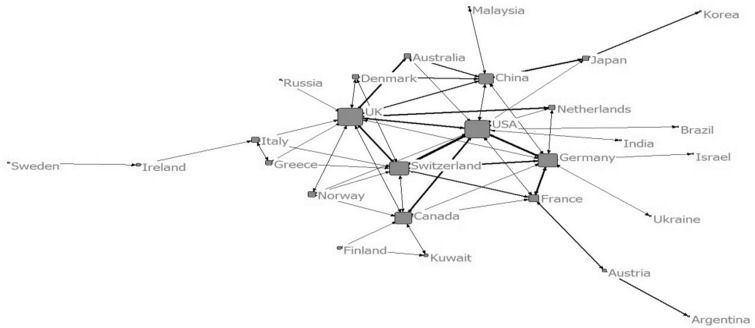
Collaboration among countries (2000–04)

**Figure 5:**
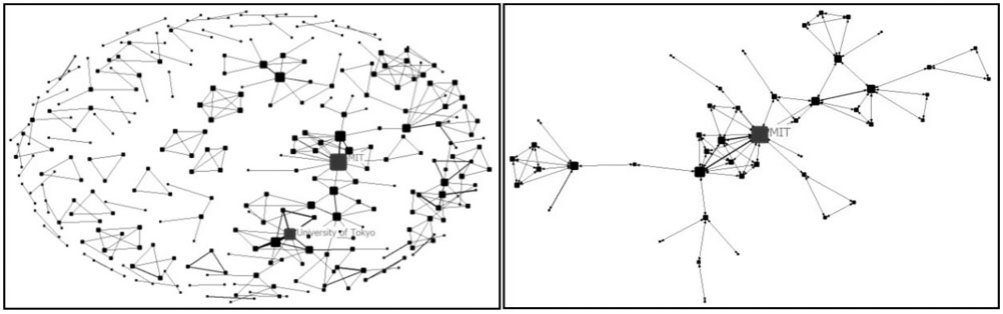
All institutions collaboration network (left) and the biggest collaboration team (right) (2000–04)

**Figure 6:**
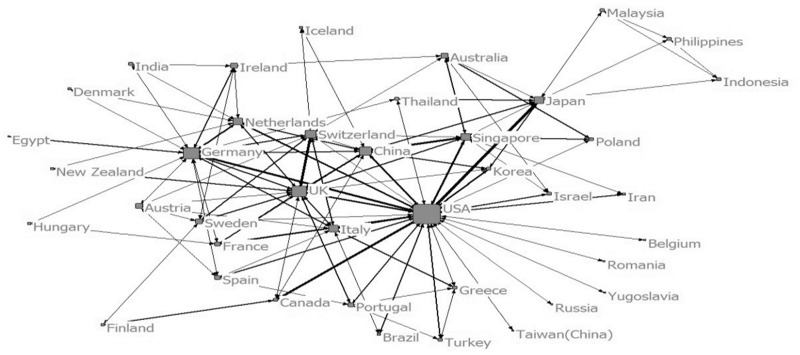
Collaboration among countries (2005–09)

**Figure 7:**
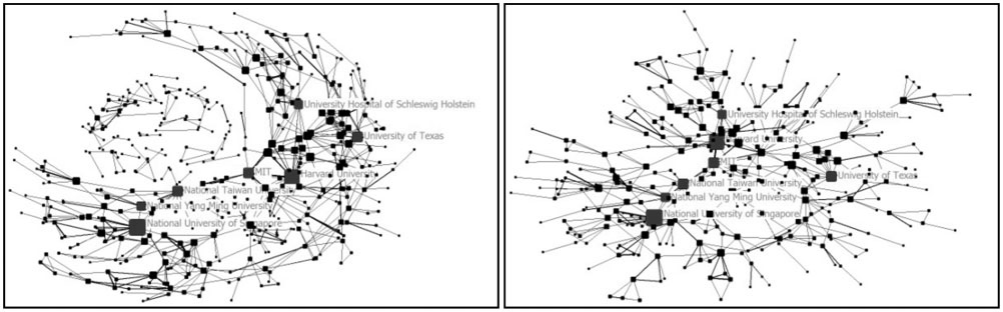
Top 500 institutions collaboration network (left) and the biggest collaboration team (right) (2005–09)

**Figure 8:**
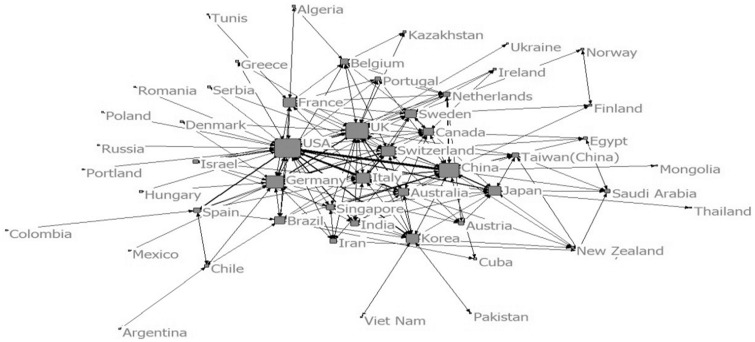
Collaboration among countries (2010–14)

**Figure 9:**
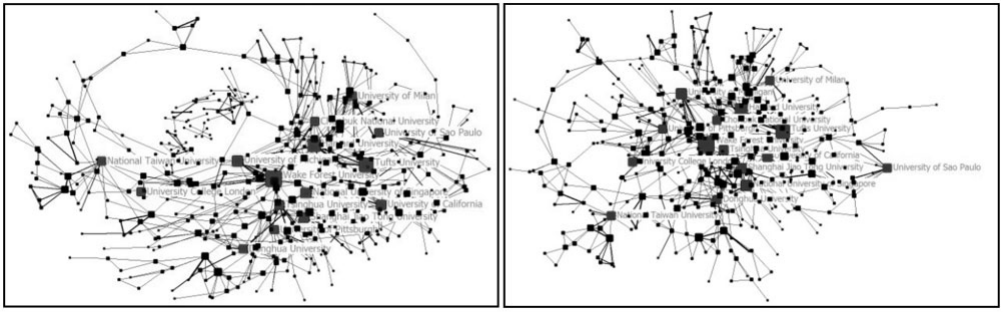
Top 500 institutions collaboration network (left) and the biggest collaboration team (right) (2010–14)

To understand the evolutionary process of collaboration, the map of collaboration network among countries and institutions were drawn by four stages according the address information of the 1158 papers with collaboration (as shown from figs 2 to 9). Each node presents a country/institution. The bigger the node is, the more collaborating partners it has. The line indicates collaboration relationship between the nodes it connected. The thickness of the line translates to the frequency of collaboration between the two countries/institutions.

In addition, this paper also calculated the structure indicators of social collaboration network in each period (as shown in Table 3 and Table 4). ‘Sample size’ refers to the total number of papers with collaboration. ‘Network size’ means the total number of countries/institutions which cooperated with others. **‘**Network tie’ is the number of connections among countries/institutions. ‘Network frequency’ is defined as the total collaboration times among all countries/institutions. ‘Network density’ is the ratio of network tie and its maximal possible value [19].

**Table 3:** Structure indicators of social collaboration network among countries

	Before 1999	2000–04	2005–09	2010–14
Sample size	10	43	145	242
Network size	10	27	41	51
Network ties	10	51	104	163
Network Frequency	12	83	200	348
Network density	0.222	0.145	0.127	0.128
Network centrality	0.625	0.540	0.526	0.520

**Table 4:** Structure indicators of social collaboration network among institutions

	Before 1999	2000–04	2005–09	2010–14
Sample size	41	178	484	855
Network size	80	304	820	1399
Network ties	66	334	617	737
Network Frequency	74	385	734	920
Network density	0.021	0.007	0.005	0.006
Network centrality	0.513	0.503	0.502	0.502

Before 1999, there were 10 papers which involved collaboration by 10 countries. The number of cooperating institutions was minimal and the network tie was only 66. Only a few countries were involved in the international collaboration. Germany and USA were central nodes of the networks. Harvard University was the central node of the largest collaboration team which only involved six institutions. This was the ‘scattered period’ when there was lack of collaboration.

In the period of 2000–04, more countries and institutions were involved in the collaboration network. The network size, network ties and network frequency all increased. USA and UK were the core countries of collaboration network. The largest collaboration team involved 46 institutions and Massachusetts Institute of Technology (MIT) was the one with the largest centrality. This was the ‘transitional period’.

In the period of 2005–09, the sample size, network size, network ties and network frequency further increased. A total of 41 countries and 820 institutions were involved in the collaboration. USA, Germany, UK were core countries in the network. The largest collaboration group was made up by 306 institutions. It was an out diffusion net made up by several larger nodes and a number of smaller nodes branching from the larger ones. Harvard University, National University of Singapore, University of Texas (USA), National Yang Ming University (Taiwan, China), MIT, National Taiwan University (Taiwan, China) and University Hospital of Schleswig Holstein (Germany) were institutions with high centralities. In this period, the collaboration was a ‘wild contact type’.

From 2010 to 2014, the sample size, network size, network ties and network frequency kept growing. 51 countries and 1399 institutions had collaborated with others. USA, UK and China were the countries with highest centralities. The largest collaboration group was consisted of 386 institutions. Wake Forest University, Harvard University, Tufts University, National University of Singapore, University of Michigan (USA), University of California (USA), Shanghai Jiao Tong University, Tsinghua University, University of Milan (Italy), University of Pittsburgh, University College London (UK), Chonbuk National University (South Korea), University of San Paulo (USA), Donghua University (China), National Taiwan University were institutions with high centralities. In this period, a big circle network of collaboration formed. It can be called ‘centralized type’.

## Discussion

At present there are only a few English papers researched on ADRIMD literatures. As far as ADRIMD literatures are concerned, eight databases (PubMed, ScienceDirect, Web of Science, EBSCO, SpringerLink, Engineering Index, BIOSIS Preview and ProQuest Dissertations and Theses) were searched, only two English papers were found, Wang and Li [20] presented a bibliometric analysis of ADRIMD literature from the mainland of China (analyzing data such as publication year, journal preference, authors’ geographic location, research topics and core expertise to predict the research trends and Wang and Li [21] analysed and summarized issues of intellectual property involved in ADRIMD. However, the collaboration relationship of these institutions was not investigated. Therefore, this paper focuses on the collaboration network based on the information of relevant English literature. The results show that the collaboration network in ADRIMD field has evolved from scattered to single-core dominated, and then to a core-edge one. Due to the growth of network frequency, network size, and the size of largest collaboration group, it can be inferred that for ADRIMD field, knowledge has been exchanged more frequently; the collaboration has been extended globally; and the network has become more interconnected.

Network density is used to reflect the closeness of nodes. The higher the density is, the closer the collaboration is. It is beneficial to facilitate information exchange and communication. On the contrary, smaller density goes against the spread and share of information in the network. From period 1–4, the network density among countries/institutions decreased, which means the closeness of the relationship of information exchange among countries/institutions has become lower. This is probably because the network size has increased rapidly, reducing previously close collaboration. But from another perspective, high density contributes to high knowledge homogenization of each node, which might be an obstacle of generate new ideas [22]. Therefore, reduction of the network density may be more beneficial for researchers, to spur creativity and innovation [23, 24]. The centrality of both the collaboration network among countries and among institutions reduced over the periods of time, which indicates reducing centralization. Over time, there were more countries and institutions joining the collaboration network and playing important roles in the network, which is good for information and resources exchange. In such kind of network, the reciprocity is better and the probability of creation is higher.

It is worth noting that developed countries, such as USA, UK and Germany, were consistently the core nodes of the collaboration network throughout the earlier periods. This can be attributed to their strong economic strength and huge research investments. Nevertheless, during the fourth period, it can be observe that the situation is gearing towards a change. China, as a vastly developing country, has become one of the core nodes in the collaboration network during the fourth period, ever since the Chinese government and scientists have placed a great importance on this area. For instance, the ‘national medium- and long-term program for science and technology development (2006–20): an outline’ propels biotechnology as one of the five key strategies of science and technology development [25]. A lot of efforts on cultivating talents, importing elites, increasing research input, participating and hosting international conferences, and establishing relationship with other countries on scientific research, had been carried out to improve the development of tissue engineering technology in China.

## Conclusion

At present, social division of labor has more and more specific, while knowledge density becomes higher over time. Collaboration is therefore very significantly important for information and resources sharing to create new thoughts, increase academic influence and enhance scientific research efficiency. Collaborative research has become a main pattern of scientific research [26].

In conclusion, from the findings of this study, English literatures of ADRIMD completed by different institutions are still scarce. The interaction among different countries can still be strengthened. The institutions in the edge of the network should enhance their collaboration and share their achievements with others especially with the ones in the center of the network which can help them to gain more information and resources. It is necessary for the developing countries which lacks collaboration to make more efforts to join the global scientific collaboration. 

## References

[1] Langer R Vacanti JP. Tissue engineering. Science 1993;260:920–6. 8493529 10.1126/science.8493529

[2] Kamel RA Ong JF Eriksson E Tissue engineering of skin. J Am Coll Surg 2013;217:533–55. 23816384 10.1016/j.jamcollsurg.2013.03.027

[3] de Vries RBM Oerlemans A Trommelmans L Ethical aspects of tissue engineering: a review. Tissue Eng Part B Rev 2008;14:367–75. 18834330 10.1089/ten.teb.2008.0199

[4] Fu Y Niu WY Wang YL Authors’ cooperation network analysis in the field of science of science. Sci Res Manage 2009;30:41–6.

[5] Dieter V. Social network analysis as a tool for research policy. Negl Trop Dis 2015;10:1–3. 10.1371/journal.pntd.0004266 PMC4697811 26720414

[6] Bollen J Van de Sompel H Hagberg A A principal component analysis of 39 scientific impact measures. PLoS One 2009;4:1–11. 10.1371/journal.pone.0006022 PMC2699100 19562078

[7] Luo JD. Social Network Analysis. Beijing: Social Sciences Academic Press, 2005.

[8] Yan Q. A review of the influence maximization problem in social network. Comput Eng Sci 2015;37:263–8.

[9] Liu J. Introduction of Social Network Analysis. Beijing: Social Sciences Academic Press, 2004.

[10] Morel CM Serruya SJ Penna GO Co-authorship network analysis: a powerful tool for strategic planning of research, development and capacity building programs on neglected diseases. Negl Trop Dis 2009;3:10.1371/journal.pntd.0000501 PMC2721762 19688044

[11] Long JC Hibbert P Braithwaite J. Structuring successful collaboration: a longitudinal social network analysis of a translational research network. Implement Sci 2016;11:19. 26864452 10.1186/s13012-016-0381-y PMC4750242

[12] Okamoto J. Scientific collaboration and team science: a social network analysis of the centers for population health and health disparities. Transl Behav Med 2015;5:12–23. 25729449 10.1007/s13142-014-0280-1 PMC4332906

[13] You H Ni J Barber M China's landscape in oncology drug research: perspectives from research collaboration networks. Chin J Cancer Res 2015;27:138–47. 25937775 10.3978/j.issn.1000-9604.2015.04.05 PMC4409971

[14] Petrescu-Prahova M Belza B Leith K. Using social network analysis to assess mentorship and collaboration in a public health network. Prev Chronic Dis 2015;12: E130. 10.5888/pcd12.150103 PMC4565512 26292061

[15] HouX N Hao YF Cao J Scientific collaboration in Chinese nursing research. Comput Inform Nurs 2016;34:47–54. 26479046 10.1097/CIN.0000000000000181

[16] Uddin S Kelaher M Srinivasan U. A framework for administrative claim data to explore healthcare coordination and collaboration. Aust Health Rev 2015 doi:10.1071/AH15058. 10.1071/AH15058 26567767

[17] Wu Y Duan Z. Social network analysis of international scientific collaboration on psychiatry research. Int J Ment Health Syst 2015;9:2. 25598839 10.1186/1752-4458-9-2 PMC4297364

[18] Kofia V Isserlin R Buchan AM Social network: a Cytoscape app for visualizing co-authorship networks. Fac 1000 Res 2015;4:481. 10.12688/f1000research.6804.1 PMC4760270 26949516

[19] Luo JD. Social Network Analysis (2nd edn). Beijing: Social Sciences Academic Press, 2010.

[20] Wang HM Li FY. Bibliometric analysis of the literature from the mainland of China on animal-derived regenerative implantable medical devices. Front Mater Sci 2014;8:403–8.

[21] Wang HM Li CY. Analysis of intellectual properties on animal-derived regenerative, implantable medical devices. Regen Biomater 2016;3:25–32. 26816653 10.1093/rb/rbv021 PMC4723278

[22] Peng H. A Study On the Impacts of Team Cooperation Networks On Knowledge Creation. Huazhong University of Science & Technology, 2010.

[23] Zhao YD Zhou C. Analysis on the cooperation of Chinese Scientists: on the perspective of individual network. Stud Sci Sci 2011;7:999–1006.

[24] Liu CF. Collaboration network analysis of scientific research based on the 973 Scheme. Sci Sci Manage Sci Technol 2013;34:14–21.

[25] Ministry of Science and Technology of Peoples’ Republic of China. The National Medium- and Long-Term Program for Science and Technology Development (2006–2020): An Outline [OB/OL][2006-02-09]. http://www.most.gov.cn/kjgh/kjghzcq/ (5 December 2014, data last accessed).

[26] Xie CX. The Function and Bibliometric Analysis of the Scientific Research Collaboration. Beijing: China Social Sciences Press, 2010.

